# A protocol to clinically evaluate liquid biopsies as a tool to speed up diagnosis of children and young adults with aggressive infection-related lymphoma in East Africa “(AI-REAL)”

**DOI:** 10.1186/s12885-022-09553-w

**Published:** 2022-05-02

**Authors:** Ismail D. Legason, Martin D. Ogwang, Clara Chamba, Elifuraha Mkwizu, Claire El Mouden, Hadija Mwinula, Lulu Chirande, Anna Schuh, Faraja Chiwanga

**Affiliations:** 1 AI-REAL Study, St Mary’s Hospital Lacor, Gulu& African Field Epidemiology Network, 180, Gulu-Uganda. African Field Epidemiology Network, 12874 Kampala, Uganda; 2 AI-REAL Study, Muhimbili University of Health and Allied Sciences, Dar es Salam, Tanzania; 3 AI-REAL Study, Kilimanjaro Christian Medical Center, Moshi, Tanzania; 4 Kilimanjaro Christian Medical University College, Moshi, Tanzania; 5 AI-REAL Study, Muhimbili National Hospital, Dar es Salaam, Tanzania; 6 Molecular Diagnostic Center, Department of Oncology, University of Oxford, Oxford, UK

**Keywords:** Liquid biopsy, ctDNA, Histopathology, EBV-driven lymphoma, Protocol, East Africa

## Abstract

**Background:**

The capacity for invasive tissue biopsies followed by histopathology diagnosis in sub-Saharan Africa is severely limited. Consequently, many cancer patients are diagnosed late and outcomes are poor. Here, we propose to evaluate circulating tumour (ct) DNA analysis (“liquid biopsy”), a less invasive and faster approach to diagnose endemic EBV-driven lymphomas (EBVL) in East Africa.

**Methods:**

We will evaluate the clinical utility of an already validated ctDNA test prospectively in a head-to-head comparison against histopathology. The primary endpoint is the time from presentation to the specialist centre to a final diagnosis of EBV- Lymphoma. Secondary endpoints include the sensitivity and specificity of liquid biopsy and health economic benefits over histopathology. One hundred forty-six patients will be recruited over 18 months. Patients will be eligible if they are 3–30 years of age and have provided written consent or assent as per IRB guidelines. Tissue and venous blood samples will be processed as per established protocols. Clinical data will be captured securely and in real-time into a REDCap database. The time from presentation to diagnosis will be documented. The sensitivity and specificity of the methods can be estimated within 5% error margin with 95% confidence level using 73 cases and 73 controls. Health-economic assessment will include micro-costing of ctDNA test and histopathology. All results will be reviewed in a multidisciplinary tumour board.

**Discussion:**

The study evaluates the clinical utility of ctDNA in improving the speed of diagnostic pathways for EBVL in sub-Saharan Africa. Our results would provide proof-of-principle that ctDNA can be used as a diagnostic tool in areas without access to regular pathology, that transfer of the tool is feasible, and that it leads to an earlier and faster diagnosis. The potential clinical and economic impact of this proposal is thus significant. If successful, this study will provide appropriate, and cost-effective diagnostic tools that will promote earlier diagnosis of EBVL and potentially other cancers in countries with restricted healthcare resources.

**Trial registration:**

Pan African Clinical Trials Registry: PACTR202204822312651, registered on 14th-April-2022.

## Background

Epstein Barr virus-driven lymphomas (EBVL) account for nearly 10% of cancer diagnoses in sub-Saharan Africa [1–3]. They disproportionally affect children and young adults and are invariably fatal without treatment [4, 5]. Importantly, 90% of patients with infection-related lymphomas can be cured provided they are diagnosed early and that the precise lymphoma subtype is identified [6, 7]. However, mortality in the region is very high, with an average 4-year survival rate estimated at 44% for endemic Burkitt Lymphoma in Uganda [8].

Despite established diagnostic pathways, treatments, and improved outcomes in high-income countries, diagnostics, treatment, and supportive care are severely limited in SSA, and outcomes are poor [9–11]. Tissue diagnosis in particular poses a significant challenge with a single pathologist being responsible for millions of patients within a region [12, 13]. Even though tissue biopsy is the gold standard for diagnosis, obtaining prompt results is a major hurdle [14]. Samples are often transported to a tertiary facility within the country or even overseas for reporting.

Research studies mostly using international haematopathology review have demonstrated that the most common aggressive childhood lymphomas are infection-related: endemic Burkitt lymphoma (BL, EBV), Hodgkin’s lymphoma (HL; EBV, HIV), acute lymphoblastic lymphoma (LBL), and diffuse large B-cell lymphoma (DLBCL; HIV, EBV ) [4, 10, 15]. Lymphomas can mimic other conditions (tuberculosis, leishmaniasis) or be associated with infections (EBV, malaria) that are common in sub-Saharan Africa [16]. This further contributes to the challenge of making a diagnosis of lymphoma correctly without histopathology.

To tackle some of the aforementioned challenges, we established a partnership with Muhimbili National Hospital (MNH), Muhimbili University of Health and Allied Sciences (MUHAS), Kilimanjaro Christian Medical Centre (KCMC), the Tanzanian Paediatric Oncology Network, African Field Epidemiology Network (AFENET), and the Epidemiology of Burkitt Lymphoma in East-African Children and Minors (EMBLEM) site at St Mary’s Hospital Lacor in Uganda and the Oxford Molecular Diagnostics Centre in the UK. The objective of this partnership is to evaluate a novel diagnostic test using cell-free circulating tumour DNA (ctDNA) that has the potential to allow early and precise diagnosis of lymphomas in resource-limited settings. This tool promises to allow the diagnosis of lymphoma from blood and to provide genetic information to aid with the accurate identification of lymphoma subtypes. We, therefore, hypothesize that our approach to the analysis of ctDNA will speed up the diagnosis of children and young adults with EBVL and that this would increase the number of children treated early ultimately leading to increased overall survival.

Unlike standard invasive biopsies, ctDNA sequencing uses a small sample of the patient’s blood. As such it offers a promising non-invasive tool to evaluate tumour heterogeneity permitting multiple sampling and monitoring of treatment response [17]. Processes such as apoptosis, tissue necrosis, and possibly active secretion by cancerous and normal cells are thought to release nucleic acids (short-double stranded DNA fragments) into circulation [18]. These fragments usually have a short half-life in circulation, in part because of rapid hepatic and renal clearance [19]. Therefore, ctDNA reflects a real-time genetic signature of the tumour.

## Methods

### Aim of the study

To improve diagnosis of childhood and young adult lymphomas in sub-Saharan Africa by making it faster using liquid biopsy.

### Specific objectives

#### Primary objective

To evaluate non-invasive cell-free circulating tumor DNA (ctDNA) detection as a diagnostic screening tool for suspected lymphoma by comparing it to gold standard invasive biopsy followed by best available local pathology. The primary endpoint is the time from presentation to the specialist centre to a final diagnosis of EBVL.

#### Secondary objectives

- To assess the sensitivity and specificity of the liquid biopsy approach for the diagnosis of other lymphomas.
- To evaluate the health-economic benefits of the novel liquid biopsy technology in an LMIC setting.

#### Exploratory objectives

- Describe the mutational landscape of paediatric and young adult EBVL in East Africa. We will document the most commonly observed genetic aberrations based on ctDNA sequencing results.
- Transfer of novel diagnostic techniques to local laboratories. We hope to develop a novel diagnostic teaching and training program for local scientists including a published curriculum, timetable, and assessments.

### Research design

This is a multicenter prospective case-control head-to-head comparison study. The clinical utility of ctDNA will be evaluated prospectively against the best local pathology with the time from the first presentation to the specialist center to the final diagnosis as the primary study endpoint.

### Study duration

We anticipate that recruitment will take 18 months and each patient will be followed up for 6 months after completion of treatment.

### Study sites

#### Muhimbili National Hospital (MNH) in Dar Es Salaam, Tanzania

Muhimbili National Hospital (MNH) is a National Referral Hospital, Research Centre, and University Teaching Hospital with a 1500 bed facility. In 2017, a total of 497 children were referred from various parts of the country to MNH and treated for different types of cancers. Of these, 90 had lymphoma (~ 20%), with 81 (~ 80%) of these confirmed BL. The Tanzanian Paediatric Oncology Network is a fully operating referral network with MNH at its centre that currently covers 40% of the country including sites where EBVL is endemic e.g., Bugando Medical Centre, Mwanza, and Sengerema in Western Tanzania.

#### Kilimanjaro Christian medical center (KCMC) in Moshi, Tanzania

KCMC is nested on the slopes of Mount Kilimanjaro in the North-Eastern part of Tanzania. It is owned by the Good Samaritan Foundation, a nonprofit organization of the Evangelical Lutheran Church. KCMC serves as a zonal referral consultant and teaching hospital for a population of 15 million people in five neighboring regions. KCMC has a 700-bed capacity offering a range of clinical sub-specialties. Cancer Care Centre (CCC) was established in December 2016 initially offering outpatient services. In January 2021 a 45-bed capacity inpatient wing with four HEPA filtered isolation rooms was commissioned. Between 2018 and 2020, a total of 108 children and young adults aged 3 to 30 received treatment at CCC. Out of these, 30(27.8%) were diagnosed and treated as cases of lymphoma.

#### St Mary’s hospital Lacor in Gulu, Uganda

Lacor hospital located in the Northern city of Gulu is a catholic founded, private not-for-profit, regional referral, and university teaching hospital. It is a 482- bed capacity with ten departments and specialised care units including paediatric oncology. Annually, the hospital treats between 100 and 150 children diagnosed with lymphomas. Burkitt lymphoma is the most common lymphoma diagnosed in the region, accounting for over half of the cases diagnosed in the year 2018. Partnering with two charity foundations; Oncology for Africa (AFRON) and Soleterre, the hospital extends psychosocial support to cancer children including accommodation and feeding during hospitalization.

### Patient eligibility

**Inclusion criteria:** Children and young adults aged 3–30 years (up to the day before their 31st birthday) suspected of having EBVL based on clinical presentation. Although a maximum duration between screening and recruitment has not been specified, given the natural history of EBVL we would anticipate that the majority of patients are recruited within 7–14 days of initial presentation. This takes into account local timelines for obtaining and reporting tissue biopsy results. All screening and eligibility assessments must be completed before the patient receives any treatment for lymphoma.

**Exclusion criteria:** Patients started on chemotherapy, and those unable, or unwilling, to provide written informed consent (and/or assent) will be excluded from the proposed study.

### Procedure for patient identification and obtaining consent

**Fig. 1:**
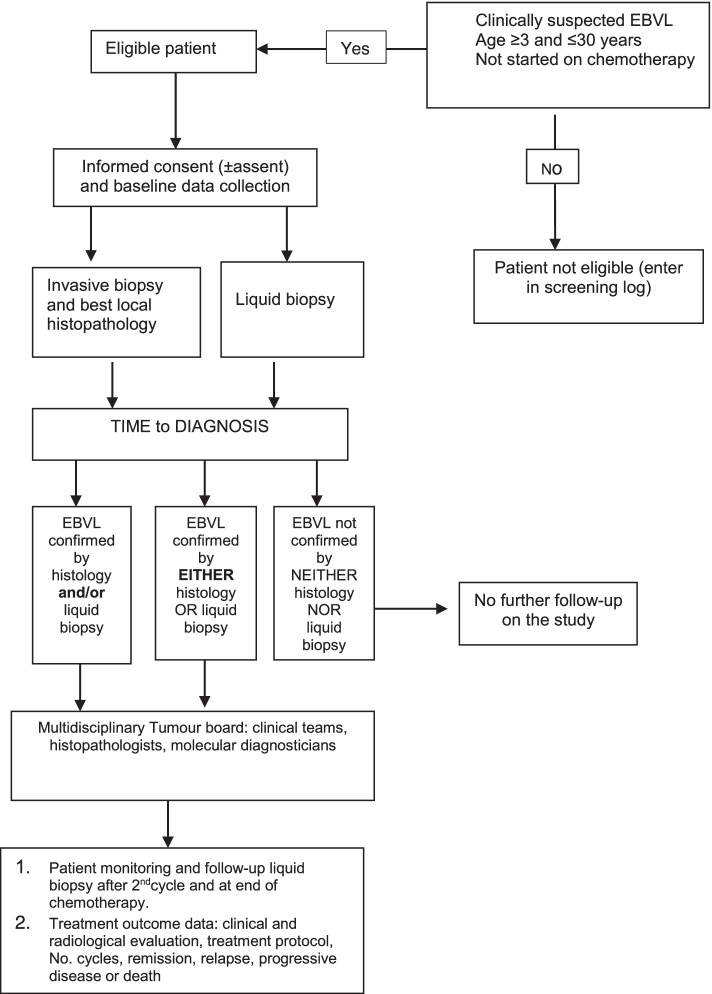
Schema for cohort observation and evaluation in the study

Children and young adults presenting with signs and symptoms suggestive of lymphoma (mostly commonly facial or abdominal swellings) will be identified through Outpatient Departments, Medicine, and Paediatric Oncology services. A study medical doctor will review the patient’s records to determine eligibility for the proposed study. Eligible patients will be approached before starting EBVL chemotherapy, for informed consent. Reasons for non-participation will be recorded for all screened patients (Fig. 1).

### Baseline data collection

#### Clinical data

An electronic database was created on Redcap Version 11.2.2 and used to collect real-time clinical data by the study staff. In summary, data collected include baseline patient demographics, general medical history, social and family history, early childhood history, and history of presenting illness. We also collect various time points from first symptoms and presentation to diagnosis and start of treatment. Specifically, we will measure the time from presentation to a treatment center to final diagnosis by either best local pathology or liquid biopsy and processing times for tissue and liquid biopsies in the laboratory.

#### Standard of care (SOC) imaging studies

SOC includes chest x-ray plus abdominal ultrasound (Lacor) or computed tomography (CT) scan (MNH, KCMC). This will depend on the site to which the patient initially presents.

#### Best local gold standard diagnosis

Open or trucut biopsy histopathological assessment that includes a limited panel of immunohistochemistry (IHC) stains and genetic analyses on the tissue as may be required. For BL, the diagnostic algorithm developed by Naresh et al. [20] is followed. For Classical Hodgkin Lymphoma (CHL), diagnosis relies on the presence of characteristic morphological features including the presence of Reed-Sternberg cells that express CD30 and often CD15. For the Nodular Lymphocyte predominant HL sub-type (NLPHL), the presence of ‘popcorn cells’ that consistently express CD20 and CD45 by IHC but are negative for CD30 and CD15 is diagnostic. For Diffuse large-B cell lymphoma (DLBCL), tumour morphology and expression of associated markers including CD10 and Bcl6 are used to classify DLBCL into germinal center B cell (GC-DLBCL) and Non-GC- DBLCL, using the classification of tumours of haematopoietic and lymphoid system [21].

#### Liquid biopsies

Three sets of samples will be collected: the first sample is taken before initiation of chemotherapy, the second sample after the second cycle of chemotherapy, and then at the end of therapy. Nine milliliters of peripheral blood will be collected into BCT®Streck tubes or Roche tubes for cfDNA analysis. Another 4 ml of blood will be collected in EDTA for isolation of PBMCs and frozen at − 70 °C / -80 °C. Both blood draws will occur at the same time as routine blood sampling. Plasma from the cell-free DNA collection tube is then double spun, aliquoted, and frozen at − 70 °C / -80 °C. Frozen plasma samples are batched and shipped from field laboratories under temperature-controlled conditions (dry ice) to a central sequencing laboratory in MUHAS in Tanzania or CPHL in Uganda for analysis.

#### DNA extraction

At the sequencing facility, cfDNA is extracted using QIAamp Circulating Nucleic Acid Kit (Qiagen, USA), as per the manufacturer’s instructions. The yield and size distribution of free-circulating DNA is measured using Qubit 3.0 Fluorometer dsDNA High Sensitivity kit (Thermo Fisher Scientific Inc. USA) and Agilent 2100 Bioanalyzer High Sensitivity DNA assays (Agilent Technologies Inc. USA) respectively.

#### Library preparation and target enrichment

The DNA libraries are prepared using the Thru PLEX Tag-Seq HV kit (Takara Bio Inc. Japan) as per the manufacturer’s instructions. The hybrid capture is performed using IDT xGen Hybridization and Wash Kit (IDT Inc. USA) protocol. Streptavidin-coated magnetic beads are used to bind and extract biotinylated probe-hybridized target DNA. This protocol includes target enrichment by PCR prior to sequencing using KAPA HiFi HotStart Ready Mix and DNA clean up using Agencourt AMPure XP PCR Purification system (Beckman Coulter, USA). The capture library products are assessed using Qubit 3.0 Fluorometer (Thermo Fisher Scientific, USA) and Agilent Bioanalyzer High Sensitivity DNA assay (Agilent Technologies Inc., USA).

#### Sequencing libraries

The target capture library pool is sequenced on the Illumina MiSeq (Illumina, CA, USA) following the manufacturer’s protocol. Our panel design includes known translocation hotspots in MYC, IGH, and IGK, previously described in BL [22], and mutation hotspots in genes known to be recurrently mutated in HL [23].

#### Bioinformatics analysis

The bioinformatics pipeline is built on the Amazon Web Services (AWS) cloud computing system. Paired-end fastq data sequenced using the Illumina MiSeq are cleaned, trimmed, clustered, and deduplicated using bespoke scripts and aligned to the human genome (hg19) using BWA-mem 2 [24]. Variants are annotated using Vardict [25], Varscan 2 [26], and Mutect 2 [27]. Filtering options for SNVs and small insertions and deletions are provided including variant allele frequency (VAF), quality, coverage, and strand bias. Translocations are called using GRIDS S [28], Aperture [29], and SViCT [30] and combined using bespoke scripts. The percentage of ctDNA is estimated using the VAF of known somatic mutations. Variants of known clinical significance including translocations will be combined into a clinical report that can be fed back to clinical scientists as well as be used for research purposes.

#### Follow-up study visits

Patients with a confirmed diagnosis of lymphoma will be followed up 3-monthly for 6 months following completion of their final cycle of chemotherapy. Information reviewed as part of patient follow-up will include medical history – current symptoms, duration of symptoms, the severity of symptoms (graded as per CTCAE v4.03), general physical assessment including assessment of the patients’ peripheries, cardiovascular system, respiratory system, abdominal examination (with documentation of findings including the presence or absence of lymphadenopathy or organomegaly) and ear-nose-throat assessment. Concomitant medications – a list of medication (including any vaccinations) taken by the patient in the 12-weeks before the presentation- will also be asked. SOC blood tests including full blood picture, urea, and electrolytes, liver function, and lactate dehydrogenase will be requested.

#### Data management

For clinical data, we will use a password-protected instance of REDCap stored on a secure server at MNH. Only essential research staff have access to the database. For targeted sequencing data, we will use a HIPAA-compliant cloud. Access will be password protected and restricted to study staff. Patients will be given a unique study ID. In line with ethical procedures, all samples will be anonymised and receive a unique sample ID before transfer to the research laboratory. This sample ID will also be used for the transfer of the data. Reports from standard-of-care tests will be linked to the study IDs by treating clinical teams who will erase patient IDs before reports are given to the research team. All identifiable information will remain at the clinical study sites. Data storage will be on an encrypted and password-secured database in the HIPAA compliant cloud.

#### Statistical analysis and sample size estimation

Sensitivity and specificity will be calculated based on a confusion matrix constructed by applying the test (liquid biopsy) and the gold standard (invasive biopsy and best local histopathology) on several cases (positive for lymphoma) and controls (negative for lymphoma) and comparing their output. We require that, for a reliable test, both metrics are at least 95%. Under this assumption, both sensitivity and specificity can be estimated within a 5% margin of error at a 95% confidence level, given a sample of 73 cases and 73 controls. By relaxing the above reliability requirement to, for example, 80% sensitivity and specificity, the same level of precision would require 246 cases and 246 controls. With 73 cases and 73 controls, on the other hand, the attainable margin of error, in this case, is slightly lower than 10%.

The performance of the test in relation to the gold standard will be assessed by testing whether the observed sensitivity or specificity is significantly lower than the expected value of 95% using the (exact) binomial test. Given 73 cases and 73 controls, we can detect an observed sensitivity or specificity at least 10% lower than the expected value of 95% with 84% power at a 95% confidence level. Detecting smaller differences between observed and expected values requires larger samples. For example, detecting a difference of only 5% with 80% power and 95% confidence would require 203 cases and the same number of controls.

Finally, assuming 73 cases and 73 controls and given the current prevalence of EBVL at 50%, we estimate that 300 patients will have to be screened to generate the number of cases and controls required for our analysis.

#### Validity of the study

To minimise sources of error and likely bias, we will ensure the following: 1) Gold standard: The liquid biopsy will be compared to the best locally available histopathology consisting of; morphological assessment of H&E stains, immunohistochemistry as described, scans of whole slide images for second opinion to allow independent review of all cases by at least two study pathologists.2) Measurement of variables: ctDNA will be extracted and sequenced in a central laboratory following standard operating procedures by well-trained laboratory scientists thus minimizing inter-laboratory variations. The laboratory will participate in external quality control schemes of next-generation sequencing. 3) Data collection and analysis: The study has uniform data collection forms (CRFs) across all sites and data is entered into the REDCap database in real-time and analysed weekly for quality metrics.

#### Ethical conduct of the study

The Investigators will ensure that this study is conducted following the principles of the Declaration of Helsinki. The protocol received an initial peer review (on the behalf of the funder) by a Scientific Review Group (SRG), a committee of scientists who have expertise in relevant scientific disciplines and current research areas.

Subsequent approvals were granted by the Oxford Tropical Research Ethics Committee (OxTREC), National Institute of Medical Research (NIMR) in Tanzania, Uganda National Council of Science and Technology (UNCST), and Lacor Hospital Institutional Research Ethics Committee. Initial REC approval was given on the 6th February 2019 (protocol V3.1), and the current protocol V3.3 was approved on 4th April 2021 via substantial amendment.

The Investigator will submit and, where necessary, obtain approval from the above parties for all amendments to the original approved documents.

#### Informed consent

Informed consent will only be sought by study delegated staff, trained in informed consent and assent including local oncology/haematology fellows in training. Participants, parents, or legal guardians must personally sign and date the latest approved version of the Informed Consent form before any study-specific procedures are performed.

Approved versions of the Participant Information Sheet and Informed Consent, written in Kiswahili, Luo, or English are given to the participants/parents and reviewed verbally. Participants are free to withdraw from the study at any time for any reason without prejudice to future care, and with no obligation to give the reason for withdrawal. Child assent is documented on a separate form written in simplified language, with space for the child to give their thumbprint, draw a smiley face, or write their name.

#### Patient confidentiality

The study staff will ensure that the participants’ anonymity is maintained. The participants will be identified only by a participant ID number on all study documents and any electronic database, except for the CRF, where participant initials may be added. All documents will be stored securely and only accessible by study staff and authorised personnel. The study will comply with the European Union’s General Data Protection Regulation (GDPR) 2018 and national regulations, which require that personal data must not be kept as identifiable data for longer than necessary for the purposes concerned.

#### Patient safety and inconvenience

This study is minimally invasive and the amount of blood drawn specifically for DNA testing is approximately 9 ml. Wherever possible, it is taken in conjunction with other tests required for evaluation and standard of care of the patients thus preventing additional inconvenience to the patient. We understand that children and young adults suspected of having lymphoma, including their families, are psychologically and physically vulnerable. Working with our partners, Soleterre in Uganda and Tumaini la Maisa (TLM) in Tanzania, we will ensure patients and their families receive adequate psychosocial support by providing both written and verbal educational materials about cancer.

#### Patient care and benefit

The study supports clinical care by offering targeted treatment with rituximab and chemotherapy to all eligible patients. If necessary, patients receive financial support to fund public travel to the hospital. In some circumstances, we provide additional diagnostic tests as advised by the patient’s doctor.

#### Study oversight and scientific advisory committee

The study is overseen by a Steering Committee (SC) composed of an independent chair, the principal investigators, an external scientist who is also part of the scientific advisory board, representatives of the funder, and the sponsor’s business unit. The SC provides oversight of scientific validity, ethical conduct, operational aspects, and finances.

#### Study monitoring and safety

An independent data monitoring committee composed of two external experts in the diagnosis and management of childhood lymphomas in Africa has been established. The committee reviews diagnostic data and MDT decisions every 6 months to establish the validity of the diagnostic process. All cases of discordance of liquid biopsy with pathology will be reviewed and their clinical management will be based on routine pathology in line with the standard of care. In addition, we will perform internal yearly horizontal and vertical audits of trial conduct and data source verification.

#### Dissemination of trial results

We will disseminate our findings in oncology, haematology, and global health journals. This will include presentations and publications at the African Organization for Research and Training in Cancer (AORTIC), the American Society of Haematology (ASH), and European Haematology Association (EHA) conferences. All institutions and funders will be acknowledged appropriately.

Our partner, AFENET works with Government ministries and universities across Africa and will provide a seamless introduction of our technology and research findings. We will hold yearly workshops with the patient and parent representatives, members of the Ugandan and Tanzanian Ministries of Health, private and public healthcare insurers and biotech companies, and NGOs. We will share results and learn from the programme and begin to develop a plan of how our technologies can be introduced sustainably into routine healthcare provision. We will have a website and quarterly newsletters to describe the progress and achievement of milestones.

#### Publication policy

AI-REAL works as an equitable collaborative consortium between investigators from 3 different countries. Authorship will be attributed purely based on contributions to the specific manuscript. By the nature of the work, this is most likely to result in 2 co-first and 2 co-last authors, each from Oxford and Africa. All other authors sign under the umbrella of “on behalf of the AI-REAL consortium to reflect their contributions during the weekly scientific meetings.

#### Final trial data set

Clinical data will be made available to the WHO cancer registry upon completion of the study. We intend to publish the sequencing data generated in a suitable public repository. We will ensure to provide the information on deposition and how to access these data in the ‘Availability of Data and Materials’ section of our publication including the permanent link or the unique identifier associated with it.

Access to the full protocol including participant-level data set and statistical codes will be granted following the final publication of the AI-REAL consortium or as part of academic collaborations.

## Outlook

Our long-term goal is not just to transfer this technology to laboratories in countries affected by BL, but also to extend the analysis of liquid biopsies to other cancers more generally. We will create capacity by developing a novel diagnostic teaching and training program for local scientists and test different delivery models in the private and public sectors starting with the National Central Public Health Laboratory in Uganda and the social enterprise spin-out from MUHAS, SEREN, that won the University of Oxford Vice-Chancellor award of Innovation 2020 and that aims to deliver diagnostic DNA services at affordable cost including early cancer screening from blood tests, diagnosis of haemoglobinopathies, SARS-CoV2 pathogen surveillance, and other inherited blood disorders.

## Data Availability

Following publication of the results, sequencing data will be made available for research purposes by request to the steering committee or lead investigator.

## Abbreviations

AI-REAL: Aggressive Infection-Related East African Lymphoma
AFENET: African Field Epidemiology Network
AFRON: Oncology for Africa
ASH: American Society for Haematology
AORTIC: African Organisation for Research and Training in Cancer
BL: Burkitt Lymphoma
BCT: Blood collection tube
CCC: Cancer Care Centre
ctDNA: Circulating tumor DNA
CI: Confidence interval
CHL: Classical Hodgkin’s lymphoma
CTCAE: Common Terminology Criteria for Adverse Events
CGH: Comparative Genomic Hybridisation
CRF: Case Research Forms
GCP: Good Clinical Practice
GDPR: General Data Protection Regulation
GC-DLBCL: Germinal center-Diffuse B-cell lymphoma
DLBCL: Diffuse Large B- Cell Lymphoma
DNA: Deoxyribonucleic acid
EBV: Epstein Barr Virus
EBVL: Epstein Barr virus-driven Lymphoma
EHA: European Haematology Association
EDTA: Ethylenediamine tetra acetic acid
FP: False Positive
FN: False Negative
GSM: Glass slide microscopy
H&E: Haematoxylin and Eosin
HL: Hodgkin lymphoma
HIPAA: Health Insurance Portability and Accountability Act
ID: Identity
IHC: Immunohistochemistry
KCMC: Kilimanjaro Christian Medical Center
MNH: Muhimbili National Hospital
MUHAS: Muhimbili University of Health and Allied Sciences
MDT: Multidisciplinary Tumour Board
NIHR: National Institute for Health Research
NIMR: National Institute for Medical Research
NLPHL: Nodular lymphocyte-Predominant Hodgkin’s lymphoma
OxTREC: Oxford Tropical Research Ethics Committee
OPD: Outpatient Department
PCR: Polymerase Chain Reaction
RIGHT: Research and Innovation for Global Health Transformation
SOC: Standard of care
SPO_2_: Spatial Oxygen saturation
SSA: Sub-Saharan Africa
TLM: Tumaini La Maisa
TP: True Positive
TN: True Negative
UNCST: Uganda National Council of Science and Technology
WHO: World Health Organization
